# ToxLex_bn: A curated dataset of bangla toxic language derived from Facebook comment

**DOI:** 10.1016/j.dib.2022.108416

**Published:** 2022-06-24

**Authors:** Mohammad Mamun Or Rashid

**Affiliations:** Bangla Language Technology Specialist, Bangladesh Computer Council & Assistant Professor, Jahangirnagar University, Dhaka, Bangladesh

**Keywords:** Cyberbullying, Online hate, Facebook Comments, Bengali slang

## Abstract

Toxic Language in social media is a newly emerging virtual disorder of human society. Detecting toxic language is an NLP task that requires a Dataset of utterances [1]. For the Bangla language, very few datasets have been developed on toxicity or similar concepts [2]. A dataset has been developed using user-generated content from Facebook and that will cover the demographic and thematic distribution of Bangla toxic language generated on the web. Therefore, 2207590 comments have been collected, annotated, and thus extract about 1959 unique bigrams as utterances, which were considered as base-entry of a toxic language dataset. The core derivatives of the dataset are bigram-based wordlists, which are annotated inductively and divided into 08 thematic classes that give some ideas on toxicity variations found in the Bengali community. These thematic classes cover political hate speech [3] and misogynist bullies dominantly. However, these thematic labels will serve as classifiers in the text classification process through machine learning. In addition to the thematic classification labels, this dataset includes some additional features such as imprecise meanings in English, IPA transliteration, real occurrences in the source pages, spelling standards, and degree of toxicity. As this is a dataset of utterance, it has de-identified and anonymous entries and no difficulties for public disclosure. Therefore, we consider this dataset as Toxic lexicon (Toxlex) as an exhaustive wordlist that is essentially a curated value-added and analyzed dataset which can be used as classifier material to detect toxicity in social media.

## Specifications Table

**Table:** 

Subject	Human-Computer Interaction
Specific subject area	Very short form (2-words) toxic, aggressive, abusive, uncivil and hateful Facebook comment data. useful for hate speech or toxic language detection and filtering.
Type of data	Text
How the data were acquired	The data was crawled from publicly open Facebook Pages. Then statistically analyzed, deduplicated, de-identified and anonymized, sorted, annotated, transcribed and thus curated by a single human.
Data format	Annotated, Analysed, Filtered Data
Description of data collection	Raw comments having Bangla texts were collected from 8 publicly open Facebook pages. Then bigrams were extracted which were occurred more than 2-times. The data were de-duplicated, anonymized and retrieve a list of unique toxic texts. Then, the occurrences into source pages were measured. At last, transcriptions, translation, spelling standards, and degree of toxicity were added by single human annotation.
Data source location	Publicly open Facebook pages
Data accessibility	Repository name: Mendeley Data Data identification number (DOI number): 10.17632/9pz8ssmc49.2 Direct link to the dataset: https://data.mendeley.com/datasets/9pz8ssmc49/2

## Value of the Data

• This dataset is useful for understanding the toxic language used in social media by Bengali users on Facebook. It reflects the behavioral pattern of the user community (mostly from Bangladesh and India) especially communal, misogynist, and sexist attitudes in digitally written form. And thus, it will reinforce toxicity detection and filtering system considering it's an NLP task.
• Machine learning and NLP practitioner can be target beneficiaries as this dataset can be used for detecting hate speech through Machine learning. Here, all bigrams will act as a toxic token and all thematic categories will be used for classification.
• Also, this dataset will be considered as gold-standard data to generate silver standard data for ML problems. Moreover, it could be useful as a benchmark to justify the Bangla toxic language-related models.
• For policy development, this dataset could be pre-cautions of cyber harms. For safer internet, entities could take necessary action to neutralize the toxicity from social media, user mind as well as from society.

## 1 Data Description

The base entry of this dataset is toxic utterance as bigram (two-words). The number of unique bigrams is 1959 and these bigrams have been written at least 1,04,747 times in the comments. These bigrams have been enlisted by processing 3,830,555 bigrams and these processed bigrams have been collected from 3,200,747 Facebook comments. After the manual annotation process, the dataset contains 1959 rows with 09 columns (*ID, Base_bigram, Topic of Discussion (Context), Meaning_(Approx.),* Transcription*_*(IPA), Thematic*_*category, Token*_*Occurrence, Unusual*_*Spelling). The definition of each column name can be in Table 1. Each row contains a base bigram with its other features such as transliterated form, spelling status, and degree of toxicity.

**Table 1:** Columns in the dataset and its descriptions.

Column	Description
*ID*	The integer representation of the unique identifier for the base entry. Example: “274”
*Base_bigram*	The string representation for each base entry. Example: “”
*Topic of Discussion (Context)*	The string representation of the linked real-life events of the base bigram. Example: “Context free”
*Meaning_(Approx.)*	The string representation of the translated form of the base bigram. Example: “Son of a slut”
Transcription*_*(IPA)	The string representation of the transliterated form in IPA of the base bigram. Example: “ kʰɑːn̪əkɪɾə put̪ə”
Thematic*_*category	The string representation of the eight thematic categories of the bigram. The considered categories are “Misogynist bully”, “Sexist & Patriarchic bully”, “Vulgar, incivility & sarcasm”, “Political hate-words”, “Religion/ communal hate-words”, “Racism on Body, Gender & Color”, “Moral Policing & Sewer, Name trolling”. Example: “Sexist & Patriarchic bully”
Token*_*Occurrence	The frequency of these words (as bigram). The number represents the total occurrences in 8 pages such as BDH, PRM, PAL, MTH, KLK, DWB, BBC, ANB. Example: 44
Unusual*_*Spelling	Indicates the status of spelling standards. ‘No’ for conventional spelling and ‘Yes’ for Typo of misspelled representation. Example: “No”
Degree*_*of*_*toxicity	Indicates the degree of toxicity. “Extreme” for the words that are extremely harmful to both the individual and the society and it is mandatory to filter out; “High toxic” for the words that hurt any other user or show disrespect “mid-toxic” sarcastic slang, troll, and comparatively less offensive but noticeable. Example: “Extreme”

**Fig. 1:**
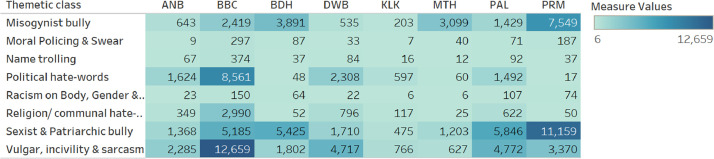
Heatmap shows thematic class/category and Source-wise occurrence of Bangla toxic words.

At the end of the annotation, 8 thematic classes/categories of the bigrams were found. through an inductive approach, that means labels or classes have been finalized after data annotation and the differences among these classes have not been identified before. The considered categories are “Misogynist bully”, “Sexist & Patriarchic bully”, “Vulgar, incivility & sarcasm”, “Political hate-words”, “Religion/ communal hate-words”, “Racism on Body, Gender & Color”, “Moral Policing & Sewer, Name trolling”. The highest number of Bigrams is 535 “Sexist & Patriarchic bully” class and the lowest number of bigrams is 12 (“Moral Policing & Sewer, Name trolling” class). In terms of occurrence, the “Sexist & Patriarchic bully” class is the most occurred class, 32371 in total and the “Racism on Body, Gender & Color” class is the less occurred at 452 in total. Fig. 1 shows a heatmap of source and class-wise occurrence metrics.

**Fig. 2:**
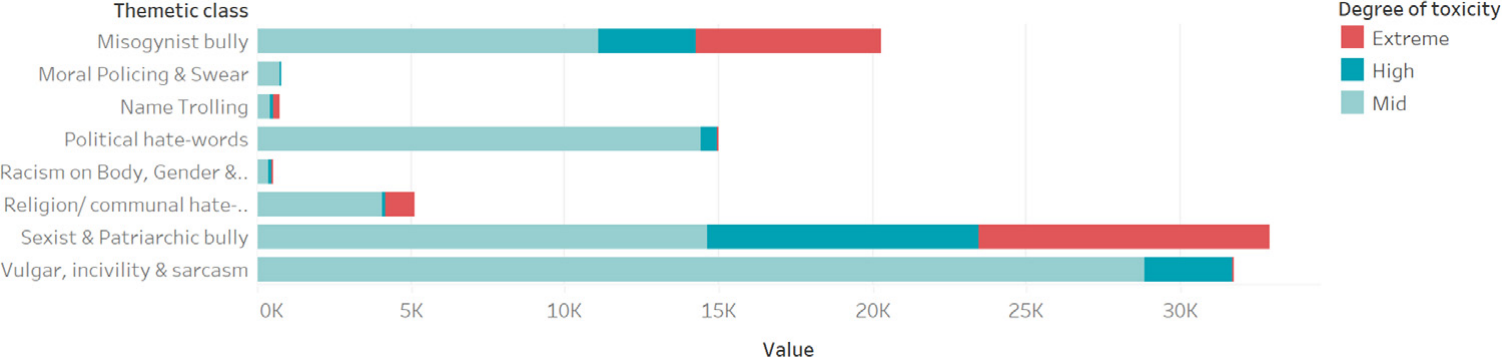
Token occurrence for each Thematic class/ category. The color shows details about the Degree of toxicity.

**Fig. 3:**
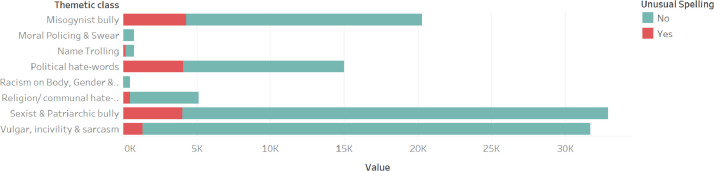
Token occurrence for each Thematic class/ category. The color shows details about Unusual Spelling.

A major addition to the dataset is the degree of toxicity. After adding three classes of degrees of toxicity, 16% of the words in the dataset were found to be extremely toxic, 15% sound high toxic, and 69% mid-toxic. Fig. 2 shows the distribution of degree of toxicity as per thematic class. Moreover, words with non-standard spelling are not omitted in this dataset. They are labeled. And as can be seen after the label, about 13% of the bigram (Fig. 3) in this dataset has been written following the highly unusual spelling of the Bengali language.

## 2 Experimental Design, Materials and Methods

This curated dataset is a derivative of raw comments. The raw data have been collected from publicly open Facebook pages. A purposeful sampling has been done to select these pages as they are the most influential Facebook pages in the Bengali language. Therefore, data were collected from 5 major news media and 3 celebrity-operated pages. The crawling time of the dataset is July 2021. and the time range of coined text data is 01 July 2020 to 30 June 2021, which means, these texts were inputted as a comment within the time range.

Raw comments have been scraped with a lot of noise, such as URLs, symbols, and emojis, Romanized Bangla, English text, Arabic text, and text by other Indian scripts. All these texts have been excluded as the current dataset contains only the bigram of Bangla Unicode text. The Romanized Bangla words have no transliteration standard and other texts written in Indian scripts are very few and thus excluded. Only Bengali Unicode texts have been kept and everything else has been removed during the cleaning process. Then, bigrams are generated from clean Bangla Unicode text and removed the bigrams which were occurred less than three times.

The bigrams are categorized into 8 'thematic' classes. Then, the translated forms of each bigram are added. While translating the nominal entities are kept intact, and the closest synonyms used in English are coined in this dataset. Then IPA as a narrow transliteration standard has been added. Consequently, two tags (Yes / No) have been added for the typo and spelling detection. Also, a degree classification of the degree of toxicity of these words has been done through Extreme / High / Mid tags. Moreover, a column expressing the topic of discussion or linked real-life events has been added to the dataset. It is found that at least 256 bigrams of the dataset have relevance to real-life events, and the rest of the bigrams have been considered context-free.

This dataset has been exported with eight columns. Among them, three have text data (Base bigram in Bangla, English Meaning, transcription IPA), three categorical data (Thematic Category, Spelling Status, Degree of toxicity), and two numeric data (ID, Token Occurrence), the first of which is continuous. And the second is discrete. This tabular data is then released in Excel format.

According to the time and source mentioned above, 3,200,747 comments have been collected as raw data. Comments, emojis, blank cell, English, and other scripts were deleted from raw as Noise. As a result, only 2,207,590 Bengali Unicode comments remained after noise-cleaning. Analyzing these comments, 61,6719 unigrams and 3830555 bigrams were found. Out of 3,830,555 bigrams, 3,75,719 were persisted which occurred more than twice, among them 2,12,377 are unique tokens. Subsequently, the unique bigrams were divided into toxic and non-toxic classes by manual annotations and 2766 toxic bigrams were extracted. Bigrams have some stop words and named entities, the number of unique Bigrams after their removal is 1959, which has been finalized for release as a dataset.

## Ethics Statements

This dataset protects the privacy of individuals. No one's personal information was taken during the data collection, moreover, no records were kept. It uses user-generated data, the names and identities of the users are not here, and sometimes the ‘*’ mark has been used to blur the identity. Who said, and whom said - both specific user identities are not kept here.

The dataset contains de-identified and anonymized very short phrase, combinations of 2-words (bigrams) from publicly open pages. As the dataset derived from the public short comment on Facebook pages, it could be made available under these Facebook guidelines and references a) What information is public? [4] b) Public content access [5] c) Comment by Graph API [6]

## CRediT Author Statement

Mohammad Mamun Or Rashid: Conceptualization, Data curation, Writing – original draft, Visualization.

## Declaration of Competing Interest

The authors declare that they have no known competing financial interests or personal relationships that could have appeared to influence the work reported in this paper.

## Data Availability

ToxLex_bn: A Curated Dataset of Bangla Toxic Language Derived from Facebook Comment (Original data) (Mendeley Data).
